# Telemedicine for the Diagnosis and Management of Age-Related Macular Degeneration: A Review

**DOI:** 10.3390/jcm11030835

**Published:** 2022-02-05

**Authors:** Grayson W. Armstrong, John B. Miller

**Affiliations:** 1 Department of Ophthalmology, Massachusetts Eye and Ear, Boston, MA 02114, USA; john_miller@meei.harvard.edu; 2 Harvard Retinal Imaging Lab, Harvard Medical School, Boston, MA 02114, USA

**Keywords:** telemedicine, ophthalmology, age-related macular degeneration

## Abstract

Use of ophthalmic telemedicine for patients with age-related macular degeneration (AMD) has shown remarkable advances over recent years. The recent COVID pandemic accelerated this transition since in-person evaluation of elderly patients at high risk for advanced AMD and severe vision loss were also at higher risk for complications from COVID infection. To date, ophthalmic telemedicine has been successfully used in remote retinal consultation by general ophthalmologists for AMD management, hybrid testing visits with both in-office testing and remote evaluation, as well as early successes in home-based remote monitoring of patients with high-risk AMD. We therefore review the current literature and evidence base related to ophthalmic telemedicine for AMD.

## 1. Introduction

Age-related macular degeneration (AMD) is the leading cause of central vision loss in the United States [1]. Patients with AMD require regular ophthalmic evaluations to monitor for disease progression towards advanced forms of AMD, namely exudative AMD or geographic atrophy. Evidence of exudative AMD warrants prompt treatment with anti-VEGF therapies to prevent vision loss, while the search continues for a successful treatment for geographic atrophy. Ophthalmic evaluation and treatment of patients with AMD pose a significant cost burden on the US health care system. AMD incidence is correlated with advancing age, and therefore the burden on the health care system will continue to increase as the population ages [2]. This places an increasing strain on ophthalmologists to adequately monitor at-risk patients, diverting resources away from regular ophthalmic care required for other eye conditions. Alternative surveillance strategies are needed to move beyond the routine office visit-based approach to AMD.

One of these alternatives is telemedicine, a strategy long deployed in other medical specialties. Telemedicine, at its core, is the use of telecommunication technologies to evaluate and treat patients separated in space and time. The provision of telemedicine can occur through various care models, including synchronous and asynchronous care and through either physician-to-patient, patient-to-physician, or physician-to-physician communication. Additionally, telemedicine also employs the use of remote patient monitoring, whereby patients are remotely monitored with medical devices capable of feeding real-time clinical data to care teams in an effort to support clinical decision making, or through the use of in-person ophthalmic imaging performed by non-physicians and subsequent remote retinal physician review, termed ‘hybrid telemedicine’. Ophthalmic telemedicine holds the potential of improving patient access to care, improving efficiency of clinical screening, and decreasing healthcare costs by allowing physicians to remotely evaluate patients. This may allow ophthalmologists to spend more in-person time with patients requiring advanced management, procedures, or surgery. The use of telemedicine for AMD has grown in recent years, holding promise for patients at risk of vision loss due to exudation or geographic atrophy secondary to AMD.

The COVID pandemic forced governments and health care facilities to severely restrict person-to-person interactions for the risk of viral transmission. This left healthcare providers grappling with the challenge of providing ongoing care to patients at a distance. The result was severely reduced access for ophthalmology patients. In order to overcome the limitations placed on in-person examination, innovation within ophthalmic telemedicine advanced at rapid speeds to allow ophthalmologists to provide ongoing care to their patients, including patients with AMD [3,4,5,6].

In this article, we will review the impact of COVID on the volume of visits for patients with AMD during the COVID pandemic, as well as advances in the use of telemedicine for the provision of care to patients with AMD. We will also comment on potential future applications of telemedicine in AMD.

## 2. COVID Impact on Retina and AMD

As the COVID pandemic unfolded and the risk to health was made evident, the American Academy of Ophthalmology responded by asking ophthalmologists to cease all non-urgent care in order to prevent disease spread [7]. Many ophthalmologists complied, with a resultant decline in access for patients at risk of vision loss. This left many patients without access to necessary eye care.

Reports of declining patient visits as a result of the pandemic shed light on the drastic change in access to eye care. In Milan, Italy, there was a 75.2% reduction in in-person visits to a retina clinic during a three-month period at the beginning of the pandemic [4]. While patients with AMD comprised the majority of visits both before the pandemic and during the height of the pandemic, there was still a 79.9% reduction in patient visits for evaluation and management of AMD. Prior to the pandemic, AMD patients made up 51.1% of clinic visits, while AMD patients accounted for only 42.7% of clinic visits during the pandemic. Additionally, the average age of patients presenting during the pandemic was lower than during the pre-pandemic period (66.7 years and 71.4 years, respectively). The report concluded that outpatient care in older patients, who are more likely affected by AMD, was more significantly impacted by the COVID pandemic.

There has also been a reported decline in the number of patients who presented to the clinic for intravitreal injections [4]. In Milan during the pandemic, there was a 53.9% reduction in the number of patients presenting for injections, which resulted in a 53.6% decline in the number of intraocular injections administered. The vast majority of intravitreal injections administered during the pandemic were anti-VEGF agents (94.5%), and the majority of injections were for the treatment of AMD (67.2%). Notably, the reduction in non-anti-VEGF therapies was more drastic than for anti-VEGF therapies in this study. The authors did not comment specifically on the preferred treatment method during the pandemic, such as treat-and-extend, fixed regimen, or otherwise.

A report out of Israel also reported a drastic decline in the number of intravitreal injections of anti-VEGF agents during the first month of the pandemic [5]. Compared to 2019, there was a 36% drop in the administration of anti-VEGF agents. After modeling for the overall rise in anti-VEGF injections year over year and predicting the expected number of injections that would have been performed were it not for the pandemic, there was over a 50% reduction in the number of administered intravitreal injections. In Israel, clinic visits for intravitreal injections were still allowed, but this report shows that patients were not reporting for their scheduled procedures, increasing the risk of ocular morbidity and visual decline.

There is also evidence that patients with AMD had worse outcomes as a result of the changes highlighted above. The same group out of Milan, Italy, performed a study to assess the impact of delayed care during the COVID pandemic [6]. This study showed that patients presenting during the pandemic had delayed their care by roughly 30 days compared to prior interval assessments. Visual acuity was statistically significantly worse during pandemic retina appointments compared to pre-pandemic vision levels. On optical coherence tomography (OCT), 81.2% of patients presenting during the pandemic had evidence of exudation, a statistically significant increase from pre-pandemic levels of 68.7%. Overall, this suggests that delays in ophthalmic care for AMD patients as a result of the COVID pandemic resulted in worsening vision and increased ocular morbidity.

Worse clinical outcomes were also identified among patients with newly diagnosed treatment-naïve AMD during the pandemic [8]. When comparing fifty consecutive patients evaluated during the pandemic with new-onset exudative AMD to controls from the two years prior, there was a statistically significant decline in best-corrected visual acuity for patients both at initial presentation and after 3 months of monthly anti-VEGF injections. Additionally, structural markers of disease activity were worsened during the pandemic. The height and width of subretinal hyperreflective material were statistically greater in patients presenting during the pandemic, while the height and width of subretinal fluid approached significance as well.

Overall, the above studies suggest that AMD still requires ongoing monitoring and treatment despite the moratorium on in-office visits. A delay in ophthalmic care as a result of the pandemic puts patient vision at risk. Fortunately, telemedical care for AMD patients presents a novel way to provide high-quality ophthalmic care to patients while maintaining a level of social distancing to prevent infection.

## 3. Telemedicine for AMD

Prior to the onset of the COVID pandemic, work to establish telemedical protocols for screening, diagnosis, and management of patients with AMD was ongoing. Success in ophthalmic telemedicine as applied to other retinal conditions, including diabetic retinopathy (DR) screening and retinopathy of prematurity screening, proves the utility of telemedicine to detect potentially blinding disease in at-risk populations [9]. However, there is not yet clinical consensus as to the utility of ophthalmic telemedicine in long-term remote management of patients with DR, retinopathy of prematurity, and AMD.

In order for telemedicine to be applied, research initially set out to prove that ophthalmic imaging and testing without an in-person examination is sufficient to detect patients with characteristic ophthalmic lesions associated with AMD. Stereoscopic fundus photographs, which allow retinal specialists to visualize the macula in three dimensions, have been found to be sufficient to detect patients with choroidal neovascular membranes (CNVM) resulting from AMD [10]. Notably, stereoscopic fundus photography is not often used in clinical practice due to the specialized training required to capture stereoscopic images, as well as the need for equipment to view fundus photographs in three dimensions, which is not commonly available. Non-stereoscopic two-dimensional fundus images, when compared to stereoscopic photos, have also been validated as being able to detect intermediate drusen (83–93% agreement), CNVM (94–98% agreement), and geographic atrophy (94–96% agreement) [11]. Furthermore, when comparing non-stereoscopic mydriatic color fundus photographs to in-person ophthalmic examination with the addition of fluorescein angiography, the resulting sensitivity and specificity of the fundus photographs in the detection of high-risk dry AMD and active exudative AMD was 82.1% and 79.1%, respectively [12]. Non-mydriatic retinal fundus photos also show similar results in detecting referrable AMD [13,14]. The use of OCT images collected by non-physicians for the detection of subretinal fluid and intraretinal fluid by remote retina specialists has also been assessed, with one study showing 100% agreement for subretinal fluid and 98.5% agreement for intraretinal fluid [15]. Combining OCT and color fundus photography has also been found to have benefits in detecting AMD during screening exams whereby non-ophthalmologists gather the images for remote review by a retina specialist [16]. Additionally, non-imaging-based ophthalmic testing, including Amsler grid testing and preferential hyperacuity perimetry (PHP), have been found to be useful in detecting exudative changes in patients with AMD [17]. Amongst patients at risk for exudation, changes in Amsler grid testing were found to have a positive predictive value of 18.9%, while changes in PHP yielded a 6.3% positive predictive value. Each of these tests can be deployed in the home through self-screening or home-based medical devices and could offer strategies for remote patient monitoring and bolster various telemedical models of care. Ultimately, these results suggest that imaging tools of the retina can adequately detect important clinical features of AMD. Additional advances in retinal imaging and testing, including OCT Angiography, may continue to support and improve the quality of remote telemedical evaluation of patients with AMD.

Ophthalmic telemedicine has implemented retinal imaging in screening programs for various ophthalmic diseases. Unlike telemedical screening for DR, which has consensus on positive cost savings and improved patient outcomes, population-based screening for AMD has not been found to be cost effective [18]. This may be due to the fact that telemedicine DR screening programs can focus solely on patients with a history of diabetes mellitus, and the risk of diabetic retinopathy is known to be relatively high in this population. Conversely, one of the only known risk factors for AMD is advanced age, thus broadening the population needing to be screened and limiting the number needed to treat in order to detect vision-threatening AMD. Overall, this has limited broad uptake of telemedical programs to screen for AMD.

The use of ophthalmic telemedicine for the management of patients with known AMD has also been evaluated. Evaluations of the effectiveness of telemedical evaluations of patients with AMD compared to in-person gold standards have been performed. In one study out of Spain, patients received in-office evaluation of visual acuity, fundus photography, and fundus OCT which was used to determine the need for intravitreal ranibizumab treatment; reassessment of each patient’s imaging test was performed remotely by the same two retinal specialists by randomizing the patient imaging between them [19]. Compared to in-person evaluation by the physicians, the remote evaluation resulted in the same management decisions for AMD patients with a sensitivity and specificity of 96% and 85%, respectively. Additionally, the remote evaluations took an average of 1 min and 21 s, while in-person evaluations took an average of 10 min. This highlights the potential improvement in efficiency gained by implementing telemedical evaluation and management of AMD patients.

A separate study out of England retrospectively evaluated a telemedical model whereby patients with a history of exudative AMD requiring anti-VEGF injections but with recent stability not requiring treatment underwent visual acuity testing and OCT scans of the macula during a hybrid in-person testing visit without real-time in-person physician evaluation [15]. Pilot testing of the telemedical care model, whereby two remote retinal physicians would evaluate the visual acuity and OCT, led to good interrater reliability (kappa = 0.862) in regard to decision to treat with anti-VEGF agents. The care model was continued beyond the pilot, and when vision-threatening AMD was remotely detected, treatment was initiated promptly. For patients with exudative AMD, the percentage of patients obtaining >15 letter improvement was 23.1% in the telemedical model compared to 6.9% in conventional care. The average interval time between appointments decreased to 5.3 weeks from 6.9 weeks after the implementation of the telemedical model, suggesting improved access to the ophthalmic care in the telemedicine model. Additionally, the average visit length was 47.3 min in the telemedical model compared to 71.4 min in the in-person model, reinforcing the idea of improved efficiency using telemedicine. Overall, these results suggest satisfactory clinical outcomes for virtual management of AMD patients while enabling closer monitoring and a reduction in visit length.

Concordance between in-person ophthalmic evaluations and remote evaluations of retinal photographs within a telemedical network between rural Nepal and Philadelphia, Pennsylvania, has also been assessed [20]. In the study, on-site technicians and ophthalmologists evaluated patients, and a retinal photograph was then transmitted to Philadelphia-based retinal physicians to evaluate for presence of AMD or DR. The overall rate of AMD in the Nepalese population ranged from 3.1% to 9.7% depending on the region. The retinal photographs were deemed sufficiently high quality to detect AMD and DR, and the conclusion was that telemedicine between the USA and Nepal is appropriate due to the lack of on-site specialists in rural Nepal.

Prospective randomized control trials have also assessed the effectiveness in managing AMD patients with telemedicine. In one study out of Canada, patients with either stable AMD or suspected neovascular AMD were randomized to in-person or telemedical appointments [21]. In the telemedical appointments, patients underwent best-corrected visual acuity (BCVA), intraocular pressure measurement, color fundus photography, and macular OCT at an Ocular Health Center staffed by general and community ophthalmologists. Ophthalmic examination and testing information was stored in an online database for remote review by a retina specialist, who would determine the need for an in-person visit and anti-VEGF injection. In the study, for new patient referrals diagnosed with exudative AMD, there was an extended referral-to-diagnosis time in the telemedicine group of 39.1 days compared to the in-person evaluation of 30.4 days. Patient satisfaction was similarly high in both groups. Additionally, the study followed patients previously treated for exudative AMD for evidence of recurrence through telemedical monitoring and found a prolonged detection of recurrence to treatment time in the telemedicine group (13.6 days) compared to the in-person group (0.04 days). Given the fact that in-person monitoring meant that patients saw retina specialists and could be treated on the same day, while the telemedicine model necessitated separate testing and treatment images, these findings are not surprising. However, BCVA was similar in the two groups at the end of the study. While the observed differences in the telemedical and in-person groups were statistically significant, the conclusion of the paper was that the wait times were not clinically meaningful and the outcomes support the use of telemedical models of exudative AMD diagnosis and management. While the telemedical model presented in this study resulted in extended referral-to-diagnosis times for new-onset exudative AMD and extended detection-to-treatment times for recurrent exudative AMD, improvements in clinical workflows to ensure more timely asynchronous imaging reads and expedited in-person treatment could result in increased efficacy of the telemedical model.

There are multiple examples of real-world success in the implementation of telemedical care for AMD patients. In a retrospective study out of Mayo Clinic, patients with exudative AMD were initially managed by a retina specialist but were subsequently managed by a local comprehensive ophthalmologist. In this model, the local comprehensive ophthalmologist was provided the ability to conduct a physician-to-physician telemedical consult with the retina specialist for continued treatment recommendations [22]. Over a two-year period, 200 electronic consultations were placed for 83 eyes of 59 patients. The average distance from the patient to the retina specialist was 70 miles. In 68.5% of cases, the comprehensive ophthalmologist performed an intravitreal injection and subsequently placed a consultation for further recommendations. Conversely, in 31.5% of cases, the comprehensive ophthalmologist consulted the retina specialist prior to performing any therapy. Overall, there was great compliance with the retina specialist recommendations. This is evidence that telemedical physician-to-physician consultations can be used to promote shared management of patients, can ensure subspecialty management, and can reduce travel requirements for patients, thus improving access to care.

Another example of remote telemedical consultation was implemented in Italy [23]. In the study, 94 comprehensive ophthalmologists were connected through a physician-to-physician telemedical network to 20 retina specialists. During a three-month period, the general ophthalmologists could consult the retina specialists and refer patients for treatment when appropriate. The study cited a markedly reduced time to therapy of an average of 5.5 days for the patients referred from the telemedical network compared to routine patients, who had an average delay of 28.7 days. There was also a significant improvement in visual acuity in the patients referred through the telemedical network compared to routine patients.

The COVID pandemic forced alternative ophthalmic evaluation methods that maximized social distancing, access, and efficiency. In May 2020, Miller et al. published a novel hybrid approach to the delivery of care by separating the testing component from the physician consultation of the clinic visit [24,25]. Later that same year, a research group in Israel reported their clinical experience and hybrid approach to care after the majority of their retinal physicians were forced into a 14-day quarantine [26]. During the two-week period, the retina practice implemented a hybrid visit strategy for their retina patients in order to promote social distancing, as well as allow continued care despite the decreased number of eye care providers present in the clinic. Patients previously scheduled for intravitreal injections were scheduled specific times to arrive for a hybrid telemedical visit. An initial intake nurse performed a brief subjective questionnaire, after which an OCT of the retina was performed by non-physician support staff. A quarantined retina physician would remotely review the questionnaire and OCT results, in addition to the patient’s prior medical record, and make a recommendation for treatment and follow up. For appropriate patients, a non-retina specialist ophthalmologist would perform the intravitreal injection as directed. Of the total 394 patients treated during this two-week period with an intravitreal injection, 50.5% were treated for neovascular AMD. Treatment regimens varied, with 56.1% of patients assigned to a monthly treatment regimen, while 43.9% were maintained on a treat-and-extend protocol. Unfortunately, despite these new procedures, 35% of patients scheduled for ophthalmic retinal exams did not show up for their appointments, compared to 14% prior to the onset of COVID. Patient wait times in retina clinics were not reported. Notably, nurses and support staff interacting with patients were at continued risk of COVID transmission. Future efforts to deploy automated fundus image capture not necessitating human interaction may improve the safety of similar telemedical care models.

An additional model of telemedical care is remote home-based monitoring of patients through remote patient monitoring. In this model, patients are routinely monitored with devices in the home for evidence of vision loss or structural evidence of exudation on imaging tests. Multiple devices have been trialed for home monitoring of patients, including a visual field machine, an OCT, and fundus photography [27,28,29,30,31].

Evaluations of automated home-based macular visual field and PHP testing devices have been trialed. One such device is the ForeseeHome device (Notal Vision Ltd., Tel Aviv, Israel). In one trial, 1970 participants deemed high risk for development of CNVM were screened, and a resulting 1520 participants were enrolled in the clinical trial [27]. Participants were randomized to self-testing for visual changes at home using tools such as an Amsler grid, or to daily testing with the ForeseeHome device whose results were read at a central reading center. Participants were followed for an average of 1.4 years, during which time 51 participants in the device arm and 31 participants in the self-testing arm developed CNVM. At the time of CNVM diagnosis, patients using the ForeseeHome device lost fewer letters on the ETDRS vision chart. When evaluating visual outcomes of patients who performed at-home screening at least twice per week as compared to standard care, there was an increased proportion of patients who detected exudation with a BCVA of 20/40 or better (94% versus 62%) and had a reduction in proportion of exudative events resulting in at least 15 letters of vision loss (6% versus 23%). Additionally, for patients assigned to the device arm, CNV detection was triggered by the ForeseeHome device more often (26 eyes) than by clinical exam during routine scheduled visits (14 eyes). Overall, the decline in visual acuity was less in the device arm (median: loss of 4 letters) than the self-testing arm (median: loss of 9 letters, *p* = 0.021) of the study following anti-VEGF therapy. Follow-up studies found similarly favorable results [28].

One study assessed the cost effectiveness of an automated home-based visual field machine for detection of vision loss amongst patients at high risk for conversion to exudative AMD [29]. In the economic analysis, the model resulted in a net cost to society of USD 907, a ten-year cost to the government of USD 1312, and a resulting incremental cost-effectiveness ratio of USD 35,663 per quality-adjusted life year gained. The study concluded that home monitoring of high-risk patients was cost effective, but that monitoring all patients, including those at low risk of progression for exudative AMD, was not cost effective.

Automated home-based OCT devices are also being developed. One study compared a novel prototype of a home-based sparse OCT (spOCT) with a spectral-domain OCT and found agreement in the central retinal thickness amongst sixty-two eyes of 31 participants, with an average difference of 4.52 μm [30]. Similarly compact prototype OCT devices, in one study termed the SELFF-OCT, have been studied regarding patient usability [31]. In this study, fifty-one patients with macular disease, including 39 with AMD, were enrolled and trained to use the home-based OCT device. After the training, 94.1% of patients were able to acquire a retinal image, regardless of quality, and 77% of participants were successfully able to acquire clinically gradable images of their own retinas using the device. Patient age and visual acuity did not appear to factor into inability to capture clinically gradable images.

## 4. Conclusions

Advances in telemedicine for use in AMD patients have shown remarkable progress over recent years. This holds significant promise for the provision of care to patients with AMD who may have limited health care access. This is especially true during the COVID pandemic, where in-person evaluation of patients at high risk of vision loss is not always safe or feasible. To date, telemedical care of patients with AMD has been explored through in-person imaging of patients with remote evaluation by a retina specialist, remote consultation of retinal specialists by general ophthalmologists, and through home-based remote patient monitoring of patients with high-risk AMD. Each model of care has shown promise in detecting exudative AMD in at-risk populations and ensuring patient access to subspecialists. Further studies to validate these and other models of telemedical care stand to benefit patients and providers through the preservation of patient vision, decrease in physician time spent on diagnostic and management decisions, and improved patient access to care.
